# Supplementary material for: Segmentectomy Versus Wedge Resection for Stage IA Lung Adenocarcinoma—A Population-Based Study
Source: Cancers (Basel). 2025 Mar 10;17(6):936. doi: 10.3390/cancers17060936 (PMC11940408; doi:10.3390/cancers17060936)
Supplement: Supplementary file 1 [file cancers-17-00936-s001.zip › cancers-3413937-supplementary.pdf]

# Supplements

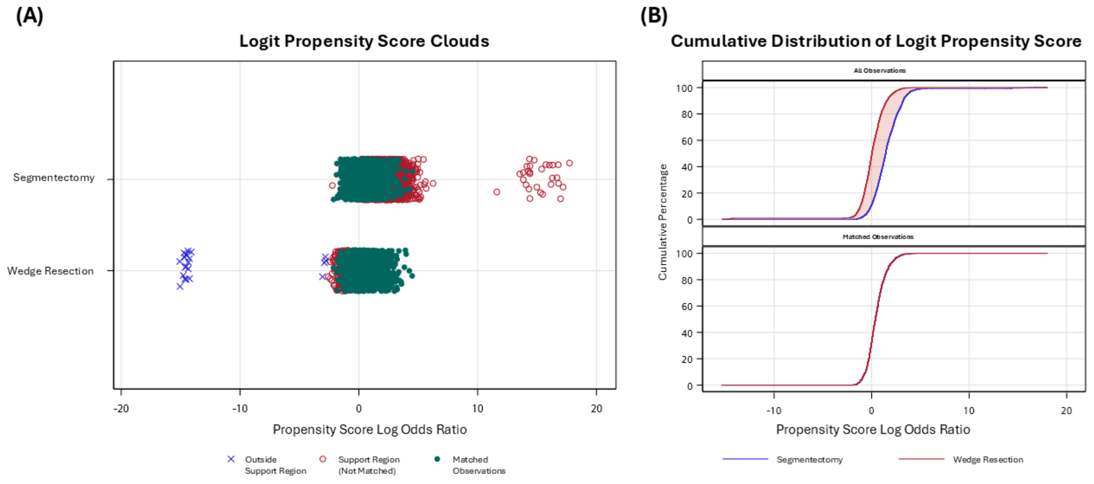


**Supplementary Figure S1.** Propensity Score Distribution and Matching. (A) Scatter plot of logit propensity scores for segmentectomy and wedge resection groups; (B) Cumulative distribution of propensity scores before and after matching.

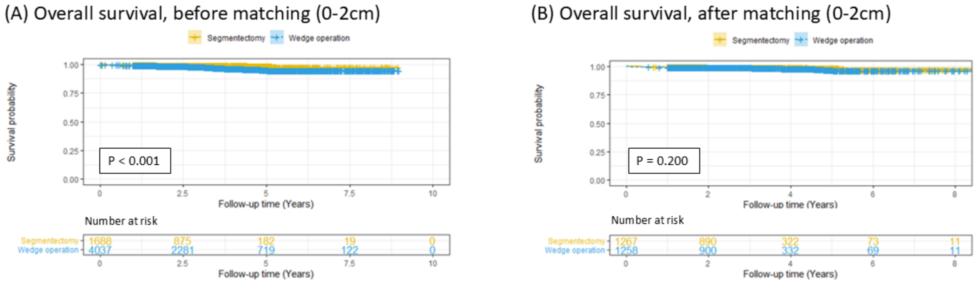


**Supplementary Figure S2**. Kaplan-Meier Analysis Before and After Propensity Score Matching for 0-2cm stage IA lung adenocarcinoma. (A) Overall survival curves prior to matching, demonstrating a significant difference (P < 0.001) between segmentectomy and wedge resection. (B) Overall survival curves following matching, showing no significant difference (P = 0.200) between the two groups.

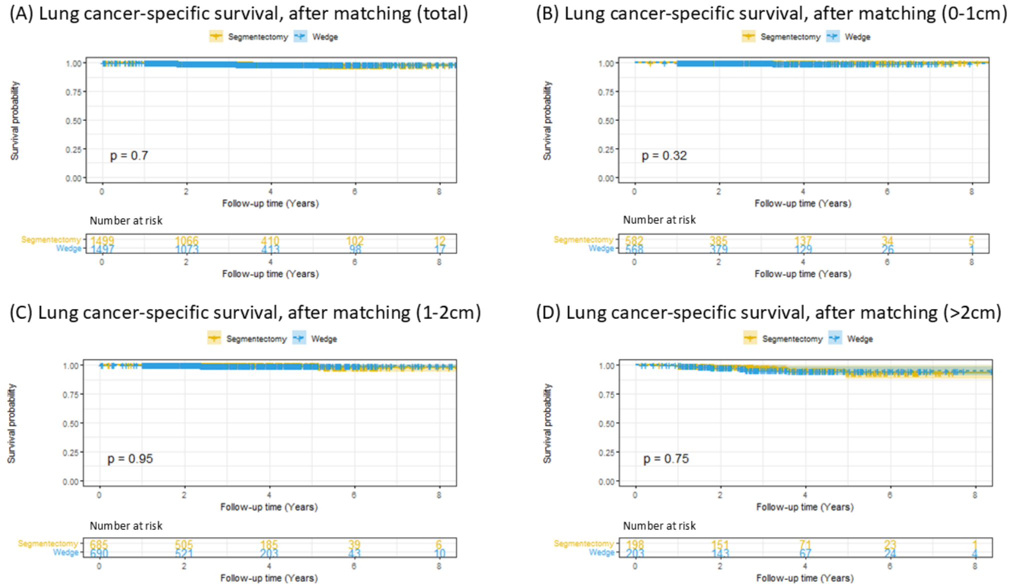


**Supplementary Figure S3.** Kaplan-Meier Analysis of Lung Cancer-Specific Survival for stage IA lung adenocarcinoma Following Propensity Score Matching, by Tumor Size. (A) Overall cohort. (B) Patients with 0-1cm tumors. (C) Patients with 1-2cm tumors. (D) Patients with tumors >2cm.
